# Comparative Genomic Analysis of *Vibrio diabolicus* and Six Taxonomic Synonyms: A First Look at the Distribution and Diversity of the Expanded Species

**DOI:** 10.3389/fmicb.2018.01893

**Published:** 2018-08-15

**Authors:** Jeffrey W. Turner, James J. Tallman, Amanda Macias, Lee J. Pinnell, Nicole C. Elledge, Danial Nasr Azadani, William B. Nilsson, Rohinee N. Paranjpye, E. V. Armbrust, Mark S. Strom

**Affiliations:** ^1^Department of Life Sciences, Texas A&M University-Corpus Christi, Corpus Christi, TX, United States; ^2^Division of Environmental and Fisheries Sciences, Northwest Fisheries Science Center, National Marine Fisheries Service, National Oceanic and Atmospheric Administration, Seattle, WA, United States; ^3^Center for Environmental Genomics, School of Oceanography, University of Washington, Seattle, WA, United States

**Keywords:** *Vibrio diabolicus*, *Vibrio antiquarius*, *Vibrio alginolyticus*, deep-sea, taxonomy, genomics, diversity

## Abstract

*Vibrio* is a diverse genus of Gammaproteobacteria autochthonous to marine environments worldwide. *Vibrio diabolicus* and *V. antiquarius* were originally isolated from deep-sea hydrothermal fields in the East Pacific Rise. These species are closely related to members of the Harveyi clade (e.g., *V. alginolyticus* and *V. parahaemolyticus*) that are commonly isolated from coastal systems. This study reports the discovery and draft genome sequence of a novel isolate (*Vibrio* sp. 939) cultured from Pacific oysters (*Crassostrea gigas*). Questions surrounding the identity of *Vibrio* sp. 939 motivated a genome-scale taxonomic analysis of the Harveyi clade. A 49-genome phylogeny based on 1,109 conserved coding sequences and a comparison of average nucleotide identity (ANI) values revealed a clear case of synonymy between *Vibrio* sp. 939, *V. diabolicus* Art-Gut C1 and CNCM I-1629, *V. antiquarius* EX25 and four *V. alginolyticus* strains (E0666, FF273, TS13, and V2). This discovery expands the *V. diabolicus* species and makes available six additional genomes for comparative genomic analyses. The distribution of the expanded species is thought to be global given the range of isolation sources (horse mackerel, seawater, sediment, dentex, oyster, artemia and polycheate) and origins (China, India, Greece, United States, East Pacific Rise, and Chile). A subsequent comparative genomic analysis of this new eight-genome subclade revealed a high degree of individual genome plasticity and a large repertoire of genes related to virulence and defense. These findings represent a significant revision to the understanding of *V. diabolicus* and *V. antiquarius* as both have long been regarded as distinct species. This first look at the expanded *V. diabolicus* subclade suggests that the distribution and diversity of this species mirrors that of other Harveyi clade species, which are notable for their ubiquity and diversity.

## Introduction

*Vibrio* is a diverse genus of Gram-negative bacteria (Thompson et al., 2004) comprising some 139 species^1^. Since Filippo Pacini first isolated *Vibrio cholerae* in 1854 (Pacini, 1854), the genus has been studied extensively as many species are readily culturable and at least 12 species are human pathogens (Chakraborty et al., 1997). *V. cholerae* and *V. parahaemolyticus* are notable in that both have been associated with pandemic disease (Karaolis et al., 1995; Matsumoto et al., 2000), and *V. vulnificus* is notable for its ability to cause primary sepsis in immunocompromised persons (Strom and Paranjpye, 2000). The genus also includes species associated with disease in commercially important fisheries and foundational species. For example, *V. harveyi* and *V. anguillarum* are pathogens of penaeid shrimp and fish, respectively (Austin and Zhang, 2006; Frans et al., 2011), and *V. coralliilyticus* and *V. shiloi* are pathogens of corals (Rosenberg et al., 2007). Yet the genus is conditionally rare (Shade et al., 2014) and many species are benign (Thompson and Polz, 2006; Thompson et al., 2006).

The geographic range of the *Vibrio* genus is extensive; representative species are commonly found in riverine, estuarine and coastal marine ecosystems worldwide (Thompson et al., 2004). Sea surface temperature can be a limiting factor, restricting growth to warmer latitudes, as many species are mesophilic (Takemura et al., 2014). However, two *Vibrio* species were originally isolated from habitats far removed from coastal ecosystems and the coincidental human host. *V. diabolicus* (strain CNCM I-1629) was first isolated from a polychaete associated with a deep-sea hydrothermal vent in the East Pacific Rise (Raguénès et al., 1997) where temperatures would support mesophilic growth. Similarly, *V. antiquarius* (strain EX25) was first isolated from seawater associated with a sulfide chimney in the East Pacific Rise (Hasan et al., 2015).

Previous reports have proposed that *V. diabolicus* CNCM I-1629 and *V. antiquarius* EX25 harbor specific genes that promote survival and persistence in hydrothermal vent ecosystems. For example, *V. diabolicus* CNCM I-1629 produces an acidic exopolysaccharide (EPS) proposed to confer resistance to high concentrations of the metallic sulfides (e.g., pyrite, chalcopyrite and sphalerite) associated with sulfide chimneys (Raguénès et al., 1997; Goudenège et al., 2014), and the *V. antiquarius* EX25 genome encodes an alkyl hydroperoxide reductase proposed to scavenge endogenous hydrogen peroxide in the deep-sea (Hasan et al., 2015) as this enzyme is also found in the deep-sea tubeworm endosymbiont *Riftia pachyptila* (Markert et al., 2007). Paradoxically, these two species also harbor hemolysins and type III secretion systems (T3SS) that are commonly regarded as virulence-associated factors in pathogenic *Vibrio* species (Goudenège et al., 2014; Hasan et al., 2015). The maintenance of these features in the deep-sea has been cited as evidence that secreted toxins are evolutionarily ancient features that may play a larger role in environmental fitness (Hasan et al., 2015).

What remains unknown is the distribution, diversity and potential virulence of these deep-sea *Vibrio* species. It is possible that both *V. diabolicus* and *V. antiquarius* exhibit a cosmopolitan distribution. A more global distribution would complement the adaptability of closely related *Vibrio* species (e.g., *V. alginolyticus* and *V. parahaemolyticus*), which have been characterized as opportunitrophs as they are capable of exploiting resources that are both spatially and temporally limited (Polz et al., 2006). Indeed, the detection of *V. antiquarius* EX25 open reading frames (ORFs) in 89 shotgun metagenomic datasets (Hasan et al., 2015) and the recent isolation of twelve *V. diabolicus* isolates in a mid-Atlantic estuary (North Inlet-Winyah Bay National Estuarine Research Reserve, SC, United States) (Klein et al., 2014) supports the hypothesis that these deep-sea *Vibrio* are more widely distributed.

During the course of a long-term, multifaceted study of *V. parahaemolyticus* (Johnson et al., 2012; Paranjpye et al., 2012; Turner et al., 2013, 2016), *Vibrio* sp. 939 was isolated from Pacific oysters (*Crassostrea gigas*) harvested from Puget Sound in the Pacific Northwest region of the United States. This study describes the discovery and draft genome sequencing of *Vibrio* sp. 939 and details efforts to identify the isolate through a comprehensive taxonomic analysis of 49 Harveyi clade genomes. Findings clearly show that *V. antiquarius* and *V. diabolicus* constitute the same species and we recommend using *V. diabolicus* as the antecedent. Findings also show that four misidentified *V. alginolyticus* strains (E0666, FF273, TS13, and V2) are synonyms of *V. diabolicus*. Moreover, the following comparative genomic analysis answers important questions about the expanded species’ distribution and diversity.

## Materials and Methods

### Sample Collection

*Vibrio* sp. 939 was isolated from Pacific oysters (*C. gigas*) harvested from Hood Canal, which is a basin of Puget Sound in Washington State (United States). The isolate was one of hundreds collected by the NOAA Northwest Fisheries Science Center (Seattle, WA, United States) during a multifaceted *V. parahaemolyticus* study (Johnson et al., 2012; Paranjpye et al., 2012; Turner et al., 2013, 2016). Briefly, oysters were scrubbed, shucked and homogenized, and presumptive *Vibrio* species were isolated by direct plating on thiosulfate-citrate-bile salts-sucrose (TCBS) agar (Becton, Dickinson and Company, Franklin Lakes, NJ, United States) overnight at 30°C. *Vibrio* sp. 939 was presumed to be *V. parahaemolyticus* based on the detection of the species-associated thermolabile hemolysin (*tlh*) gene (Bej et al., 1999), but the isolate was omitted from our previous *V. parahaemolyticus* multilocus sequence typing (MLST) study (Turner et al., 2013) as many of the MLST loci proved recalcitrant to amplification. The preliminary assignment was also called into question by two recent studies showing that the *V. parahaemolyticus*-specific *tlh* primers could also amplify sequence variants of the *tlh* gene in closely related species (Klein et al., 2014; Yáñez et al., 2015).

### Culture Conditions

Starting with a frozen cell culture (25% glycerol, v/v, -80°C), *Vibrio* sp. 939 was streaked for isolation on lysogeny broth (LB) (Fisher Scientific, Fair Lawn, NJ, United States) supplemented with 1.5% Bacto agar (Becton, Dickinson and Company, Franklin Lakes, NJ, United States) and grown overnight (18 h) at 30°C. A single isolated colony was transferred to 5 mL LB in a 15 mL BD Falcon tube (Becton, Dickinson and Company, Franklin Lakes, NJ, United States) and grown overnight (18 h) at 30°C with shaking (120 rpm) in an Excella E24 shaking incubator (Eppendorf, Hamburg, Germany).

### DNA Isolation

Bacteria in the overnight culture (1 mL) were pelleted by centrifugation (9,400 × *g*, 5 min) in an Eppendorf 5424E centrifuge (Hamburg, Germany) and washed twice with an equal volume of phosphate buffered saline (PBS). The DNA was isolated from the pelleted cells using a ChargeSwitch gDNA Mini Bacteria Kit (Invitrogen, Carlsbad, CA, United States) following the manufacturer’s protocols. The DNA was quantified and assayed for quality (*A*_260_/*A*_280_) using a BioPhotometer D30 (Eppendorf, Hamburg, Germany) and stored at -20°C.

### Genome Sequencing

Genomic DNA was sequenced with an Illumina MiSeq instrument at the New York University Genome Technology Center using paired-end chemistry (2 × 300 bp). The sequencing library was prepared using the PCR-free version of a KAPA DNA Library Preparation Kit (Kapa Biosystems, Wilmington, MA, United States). Overlapping paired reads were merged using FLASH version 1.2.11 (Magoc and Salzberg, 2011). The merged reads were trimmed of adapter sequences and low quality bases with Trim Galore! version 0.4.4^2^, which is a wrapper script for Cutadapt (Martin, 2011) and FastQC (Andrews, 2010). The optimal *k*-mer size was estimated with KmerGenie version 1.7 (Chikhi and Medvedev, 2014) and the draft genome was assembled *de novo* with Velvet version 1.2.10 (Zerbino and Birney, 2008). The assembled genome was initially annotated and inspected with the web-based RAST annotation service and SEED Viewer (Aziz et al., 2008; Overbeek et al., 2014). The final annotation was completed with the National Center for Biotechnology Information’s (NCBI) Prokaryotic Genome Annotation Pipeline (PGAP) (Klimke et al., 2009).

### Phylogenetics

The relatedness of *Vibrio* sp. 939 and 48 closely related Harveyi clade species (e.g., *V. diabolicus, V. antiquarius, V. parahaemolyticus, V. alginolyticus*, and *V. harveyi*) (**Supplementary Table S1**) was inferred by constructing a maximum-likelihood tree using a set of single-copy homologs present in all the 49 Harveyi clade genomes. The genomes were downloaded from NCBI. Single-copy homologous genes were clustered using *get_homologues* (options -M -t 49 -r EX25.gbk -e) (Contreras-Moreira and Vinuesa, 2013). The program options dictated that the homolog search was carried out with the OrthoMCL algorithm (Li et al., 2003) using the default *E*-value cutoff (1e-05) and that clustering was limited to single-copy homologs present in all 49 genomes. Additionally, the closed genome of *V. antiquarius* EX25 was used as the reference genome and clusters containing in paralogs were excluded. Homologous clusters were aligned individually with MUSCLE version 3.6 (Edgar, 2004) and alignments were trimmed to the length of the shortest sequence with trimAl version 1.4.15 (Capella-Gutierrez et al., 2009). Trimmed alignments were then filtered by length (250 bp cutoff), concatenated with the fasta manipulation tool in Galaxy (Giardine et al., 2005) and a maximum-likelihood tree was constructed with IQ-TREE version 1.5.5 (Nguyen et al., 2014) with 1,000 ultrafast bootstraps (Minh et al., 2013) using the best-fit model (GTR+I+G4) as determined by ModelFinder (Kalyaanamoorthy et al., 2017). The phylogenetic tree was annotated with FigTree version 1.4.3^3^.

### Phylogenetic Network

To visualize conflicting phylogenetic signal owing to reticulate evolutionary processes, a Neighbor-Net phylogenetic network (Bryant and Moulton, 2004) was constructed for the concatenated multilocus alignment (above) with SplitsTree4 version 4.14.4 (Huson and Bryant, 2006) using default settings and 1,000 bootstraps.

### Genome Similarity

The genomes of *V. diabolicus* Art-Gut C1 and CNCM I-1629, *Vibrio* sp. 939, *V. antiquarius* EX25 and *V. alginolyticus* 12G01, 40B, E0666, FF273, K01M1, NBRC 15630, TS13, and V2 were compared with an all-versus-all alignment using JSpeciesWS (Richter et al., 2016). JSpeciesWS is a web server implementation of the popular JSpecies Taxonomic Thresholds Program (Richter and Rosselló-Móra, 2009) that calculates the average nucleotide identity (ANI) between a query genome and a reference genome where 95–96% similarity is considered the threshold for identifying a prokaryotic species. To illustrate the sequence similarity of the eight *V. diabolicus* subclade genomes (defined in this study as *V. diabolicus* Art-Gut C1 and CNCM I-1629, *Vibrio* sp. 939, *V. antiquarius* EX25, and *V. alginolyticus* E0666, FF273, TS13, and V2), a circular blast map was constructed using the BLAST Ring Image Generator (BRIG) (Alikhan et al., 2011). For this purpose, the lower and upper identity thresholds were set at 92 and 96% (respectively) and the closed genome of *V. antiquarius* EX25 was used as the reference genome. For added contrast, the *V. alginolyticus* NBRC 15630 genome was included in the comparison. Genome attributes (e.g., genome size, %GC content, total genes, protein coding genes, RNA genes) were also calculated to compare these eight genomes.

### Pangenome Analysis

To assess the diversity of the *V. diabolicus* subclade (defined above), the pangenome of this subclade was determined by two methods. First, the pangenome was estimated with *get_homologues* using option *t* = 0 to report all homologous genes clusters and the accompanying parse_pangenome_matrix.pl script was used to call the subset of genes that constitute the core (genes present in all eight strains) and accessory (genes present in fewer than eight strains) pangenome compartments. Seeing that the *get_homologues* analysis excludes clusters with fewer than three sequences as well as non-coding sequences, a second pangenome analysis was carried out with Panseq (Laing et al., 2010) using default parameters. The Panseq software estimates the pangenome using an all-versus-all alignment of genomic regions and reports the core, accessory and pangenome. For this analysis, the core genome was defined as the genomic regions present in all eight genomes. Additionally, to capture the presence of singletons (i.e., genomic regions present in only one genome), Panseq’s Novel Region Finder was used to discover genomic regions novel to individual genomes. The novel regions were then filtered by length (10 Kb cutoff) to identify probable genomic islands (GIs). These GIs were then inspected in the web-based SEED Viewer (blastn *E*-value cutoff 1e-05) (Overbeek et al., 2014) for features linked with survival or persistence in specific habitats (e.g., deep-sea versus coastal).

### Subclade-Associated Genes

The output from the above pangenome analysis was parsed with the parse_pangenome_matrix.pl script to identify genes ubiquitous in the *V. diabolicus* subclade (*N* = 8 strains) but absent from the other 41 strains. Subclade-associated genes and their protein products were aligned individually with MUSCLE version 3.6 (Edgar, 2004) and the average sequence identity for each alignment was calculated with trimAl version 1.4.15 (Capella-Gutierrez et al., 2009).

### Metagenome Survey

To investigate the presence of *V. diabolicus* in publicly available environmental metagenomic data, the protein sequences encoded by the most conserved non-hypothetical subclade-associated genes (above) were queried against the NCBI Metagenome Protein Database (env_nr) using default blastp parameters. Database hits were declared significant if the sequence identity was comparable to the above subclade protein alignments (i.e., greater than 95%).

### Potential Virulence

The occurrence and diversity of genes related to virulence and defense was investigated using SEED subsystem feature counts implemented in the web-based SEED Viewer (above). For this purpose, the eight genomes belonging to the *V. diabolicus* subclade were inspected for proteins with significant matches (blastp with *E*-value cutoff 1e-05) to five subsystems: (1) Adhesion, (2) Toxins and Superantigens, (3) Bacteriocins and Ribosomally Synthesized Antibacterial Peptides, (4) Resistance to Antibiotics and Toxic Compounds and (5) Invasion and Intracellular Resistance. To specifically investigate enzymes conferring resistance to antibiotics, the β-lactamase proteins were aligned with MUSCLE version 3.6 (Edgar, 2004), trimmed with trimAl version 1.4.15 (Capella-Gutierrez et al., 2009) and a maximum-likelihood tree was constructed with IQ-TREE version 1.5.5 (Nguyen et al., 2014) with 1,000 ultrafast bootstraps (Minh et al., 2013) using the best-fit model (WAG) as determined by ModelFinder (Kalyaanamoorthy et al., 2017). Additionally, the β-lactamase proteins (present in all genomes) and the fosfomycin resistance proteins (present in *V. alginolyticus* TS13 and V2) were searched against the Antibiotic Resistance Genes Database (ARDB) using default parameters (blastp with *E*-value cutoff 1e-05) (Liu and Pop, 2009).

### Antibiotic Susceptibility

The production of β-lactamase was tested using Cefinase disks (Becton, Dickinson and Company, Franklin Lakes, NJ, United States) according to the manufacturer’s instructions. Susceptibility to select β-lactam antibiotics was determined as described previously (Letchumanan et al., 2015). *V. antiquarius* 939 was grown overnight (18 h) on Mueller-Hinton agar plates (Becton, Dickinson and Company, Franklin Lakes, NJ, United States) (supplemented with 1.5% NaCl) at 37°C. Overnight growth was resuspended in a filter-sterilized 0.85% NaCl solution, normalized to approximate a 0.5 McFarland standard, and fresh Mueller-Hinton agar plates were seeded with a bacterial lawn using a sterile cotton swab. Penicillin (10 units), ampicillin (10 μg), cephalothin (30 μg), and carbenicillin (100 μg) antibiotic disks (Becton, Dickinson and Company, Franklin Lakes, NJ, United States) were dispensed on the lawn and plates were incubated overnight (18 h) at 37°C. The zones of inhibition were measured and interpreted according to the Clinical and Laboratory Standards Institute (CLSI) guidelines (Clinical and Laboratory Standards Institute [CLSI], 2010). The pandemic type strain of *V. parahaemolyticus* (RIMD2210633) (Nasu et al., 2000) was tested for comparative purposes.

## Results

### Genome Sequencing

The draft genome of *Vibrio* sp. 939 was comprised of 46 contigs totaling 5,430,661 bp in length with a 44.6% GC content. The N50 of the assembly was 855,346 bp and the lengths of the smallest and largest contigs were 570 and 1,035,854 bp, respectively. The PGAP annotation detected 5,049 genes, 4,876 proteins, 112 RNAs (13 rRNA, 95 tRNA, and 4 other RNA) and 61 pseudogenes. Based on the initial evaluation of the draft genome in the SEED Viewer (the View Closest Neighbors feature), the whole-genome shotgun project was deposited as *V. antiquarius* 939 at DDBJ/ENA/GenBank under the accession number AOJB00000000.

### Phylogenetic Tree

To construct a phylogeny of the Harveyi clade, a search for homologous genes returned a total of 1,125 gene clusters shared by all 49 genomes. Sixteen of the clusters were removed by the 250 bp length filter to yield a total of 1,109 single-copy homologs. The concatenation of the homologs produced a multilocus alignment totaling 1,034,083 bp. The concatenated alignment was comprised of 595,014 constant sites and 404,946 parsimony informative sites with 217,816 distinct site patterns. The best-fit model according to the Bayesian information criterion (BIC) scores and weights was calculated as GTR+I+G4.

**FIGURE 1:**
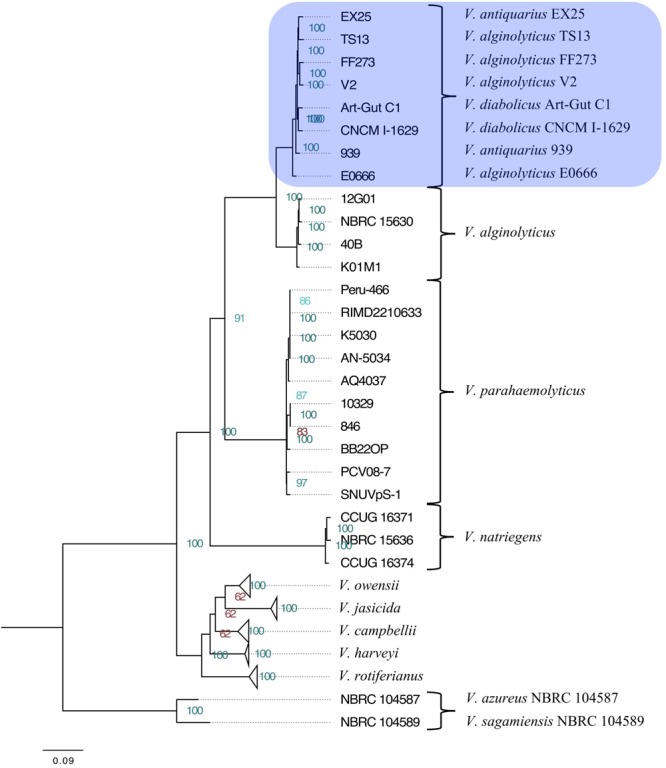
Phylogenetic tree of the *Vibrio harveyi* clade. A maximum-likelihood tree representing 49 *V. harveyi* clade genomes was inferred from the concatenated alignment of 1,109 homologous genes. Collapsed subclades represent the core *V. harveyi* clade species. The *V. diabolicus* subclade was highlighted in blue. Node labels show the bootstrap support values. Nodes with strong support (>85) were highlighted in green while nodes with less support (<85) were highlighted in red. Branch lengths represent the average number of substitutions per site. The tree was rooted to the outgroup comprised of *V. azureus* NBRC 104587 and *V. sagamiensis* NBRC 104589.

The maximum-likelihood phylogeny resolved the Harveyi clade into eleven subclades: *V. alginolyticus, V. azureus, V. campbellii, V. harveyi, V. jasicida, V. natriegens, V. owensii, V. parahaemolyticus, V. rotiferianus, V. sagamiensis*, and a mixed subclade comprised of two *V. diabolicus* strains (Art-Gut C1 and CNCM I-1629), two *V. antiquarius* strains (939 and EX25) and four *V. alginolyticus* strains (E0666, FF273, TS13, and V2) (**Figure 1**). The entirety of the tree, without collapsed branches, was included in the **Supplementary Figure S1**. This phylogeny showed strong agreement with previous whole-genome Harveyi clade phylogenies (Thompson et al., 2009; Lin et al., 2010; Urbanczyk et al., 2013, 2014, 2016; Ke et al., 2017), but unlike previous phylogenies, the aim of this phylogeny was the resolution of the non-core Harveyi subclades (e.g., *V. alginolyticus, V. antiquarius, V. diabolicus*, and *V. parahaemolyticus*). To that end, the discovery of a well-supported *V. diabolicus* subclade comprised of two *V. diabolicus* strains (Art-Gut C1 and CNCM I-1629), two *V. antiquarius* strains (939 and EX25) and four *V. alginolyticus* strains (E0666, FF273, TS13, and V2) was novel.

### Phylogenetic Network

**FIGURE 2:**
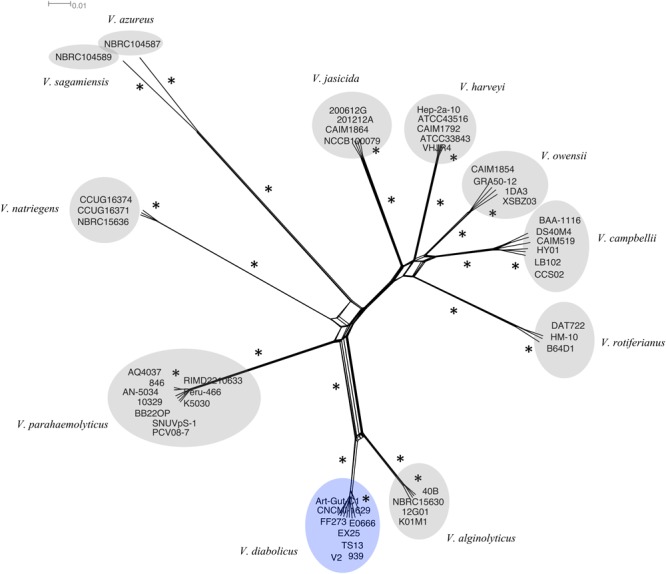
Neighbor-Net phylogenetic network of the *V. harveyi* clade. A Neighbor-net phylogenetic network representing 49 *V. harveyi* clade genomes was inferred from the concatenated alignment of 1,109 homologous genes. The *V. diabolicus* subclade was highlighted in blue while other species-specific subclades were highlighted in gray. Branches with strong bootstrap support (>85) marked with an asterisk (^∗^). The scale bar represents the number of substitutions per site.

To infer the role of reticulate evolutionary processes, a Neighbor-Net network was constructed using the same 1,109 multilocus alignment (above). The network (**Figure 2**) showed complete agreement with the phylogenetic tree. A more reticulate network was evident between the *V. diabolicus* and *V. alginolyticus* subclades. Regardless, the bootstrap analysis of the phylogenetic network showed strong support for a distinct *V. diabolicus* subclade comprised of two *V. diabolicus* strains (Art-Gut C1 and CNCM I-1629), two *V. antiquarius* strains (939 and EX25) and four *V. alginolyticus* strains (E0666, FF273, TS13, and V2).

### Genome Similarity

The relatedness of the *V. diabolicus* subclade genomes and representative genomes from its nearest phylogenetic neighbor (*V. alginolyticus*) was further analyzed by calculating pairwise ANI values (**Table 1**). Results showed that the eight genomes assigned to the *V. diabolicus* subclade (*V. diabolicus* Art-Gut C1 and CNCM I-1629, *V. antiquarius* 939 and EX25, and *V. alginolyticus* E0666, FF273, TS13, and V2) shared greater than 97% ANI. In contrast, members of this subclade shared less than 92% ANI with the representative *V. alginolyticus* genomes (i.e., *V. alginolyticus* 12G01, 40B, K0M1, and NBRC 15630).

**Table 1:** The pairwise comparison of average nucleotide identity (ANI) values between *V. diabolicus, V. antiquarius*, and *V. alginolyticus* subclade genomes.

Genome	1	2	3	4	5	6	7	8	9	10	11	12
(1) *V. diabolicus* Art-Gut C1	**^∗^**	**97.76**	**97.71**	**97.83**	**97.48**	**97.50**	**97.75**	**97.59**	91.28	91.49	91.44	91.28
(2) *V. diabolicus* CNCM I-1629	**97.89**	**^∗^**	**97.66**	**97.88**	**97.62**	**97.68**	**97.75**	**97.67**	91.38	91.47	91.50	91.47
(3) *V. antiquarius* 939	**97.65**	**97.52**	**^∗^**	**97.72**	**97.39**	**97.48**	**97.64**	**97.44**	91.27	91.41	91.43	91.33
(4) *V. antiquarius* EX25	**97.91**	**97.93**	**97.80**	**^∗^**	**97.65**	**97.80**	**97.83**	**97.74**	91.45	91.52	91.62	91.52
(5) *V. alginolyticus* E0666	**97.6**	**97.67**	**97.59**	**97.68**	**^∗^**	**97.49**	**97.52**	**97.50**	91.68	91.88	91.79	91.82
(6) *V. alginolyticus* FF273	**97.53**	**97.63**	**97.58**	**97.69**	**97.39**	**^∗^**	**97.54**	**98.08**	91.62	91.63	91.69	91.66
(7) *V. alginolyticus* TS13	**97.71**	**97.67**	**97.62**	**97.71**	**97.37**	**97.49**	**^∗^**	**97.40**	91.39	91.50	91.47	91.48
(8) *V. alginolyticus* V2	**97.68**	**97.68**	**97.57**	**97.74**	**97.44**	**98.17**	**97.54**	**^∗^**	91.62	91.67	91.69	91.71
(9) *V. alginolyticus* 12G01	91.42	91.46	91.38	91.43	91.74	91.69	91.48	91.69	**^∗^**	**98.34**	**98.33**	**98.52**
(10) *V. alginolyticus* 40B	91.59	91.53	91.49	91.47	91.86	91.77	91.57	91.72	**98.26**	**^∗^**	**98.21**	**98.31**
(11) *V. alginolyticus* K0M1	91.57	91.58	91.57	91.56	91.77	91.80	91.59	91.76	**98.25**	**98.21**	**^∗^**	**98.26**
(12) *V. alginolyticus* NBRC 15630	91.43	91.52	91.42	91.48	91.85	91.80	91.59	91.79	**98.48**	**98.30**	**98.27**	**^∗^**

*The values in bold font exceed the 95–96% threshold for species assignment. The dashed lines separate the V. diabolicus and V. alginolyticus subclades. The ^∗^ indicates 100% similarity as the genomes were compared against themselves.*

**FIGURE 3:**
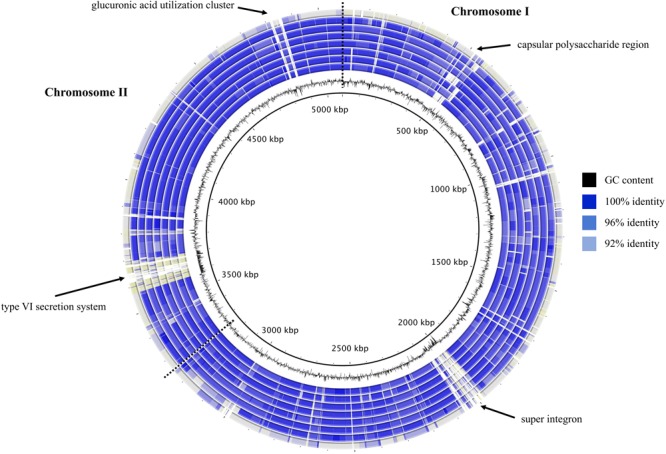
Blastmap comparison of *V. diabolicus* subclade genomes. A circular comparison of the eight *V. diabolicus* subclade genomes was based on the blastn analysis of whole genomes. Starting at the center, concentric rings show the sequence similarity between the query genome (*V. diabolicus* Art-Gut C1, *V. diabolicus* CNCM I-1629, *V. antiquarius* 939, *V. alginolyticus* TS13, *V. alginolyticus* FF273, *V. alginolyticus* V2, *V. alginolyticus* E0666, and *V. alginolyticus* NBRC 15630, respectively) and the reference genome (*V. antiquarius* EX25). Blue shading depicts regions with 100, 96, and 92% sequence similarity. Regions with less than 92% sequence similarity shown in gray. Dashed lines partition chromosomes I and II.

To visualize the synonymy in ANI values between the eight *V. diabolicus* subclade genomes, a circular blastn map was constructed (**Figure 3**). The map clearly illustrated the high degree of synonymy between the two *V. diabolicus* genomes (Art-Gut C1 and CNCM I-1629), the *V. antiquarius* 939 genome, the four *V. alginolyticus* genomes (E0666, FF273, TS13, and V2) and the reference genome (*V. antiquarius* EX25). In contrast, the non-synonymy between these genomes and *V. alginolyticus* NBRC 15630 was apparent. Notable hypervariable genomic regions labeled on the map include a capsular polysaccharide region, a super integron, a type VI secretion system (T6SS) and a glucuronic acid utilization cluster.

A more general comparison of the eight *V. diabolicus* subclade isolates (**Supplementary Table S1**) revealed that they were isolated from a range of sources (i.e., horse mackerel, seawater, sediment, dentex, oyster, artemia, and polychaete) collected from locations around the world (i.e., China, India, Greece, United States, East Pacific Rise, and Chile). The genome sizes ranged from 5,048,917 to 5,430,661 bp (*V. alginolyticus* E0666 and *V. antiquarius* 939, respectively) while the number of genes ranged from 4,572 to 5,251 (*V. alginolyticus* FF273 and *V. diabolicus* CNCM I-1629, respectively), and the GC content ranged from 44.6 to 44.9% (*V. alginolyticus* FF273 and *V. antiquarius* EX25, respectively) (**Table 2**).

**Table 2:** A summary of the attributes of the *V. diabolicus* subclade genomes: *V. diabolicus* Art-Gut C1 and CNCM I-1629, *V. antiquarius* 939 and EX25, and *V. alginolyticus* E0666, FF273, TS13 and V2.

Attribute	Art-Gut C1	CNCM I-1629	939	EX25	E0666	FF273	TS13	V2
Genome size (bp)	5,236,997	5,132,517	5,430,661	5,089,025	5,048,917	5,231,685	5,103,549	5,068,299
Level^a^	Draft	Draft	Draft	Closed	Draft	Draft	Draft	Draft
Contigs	60	29	46	2	129	179	40	33
% GC	44.8	44.8	44.6	44.9	44.8	44.6	44.7	44.8
Genes	4,843	5,251	5,049	4,778	4,616	4,705	4,858	4,572
Proteins	4,593	4,855	4,876	4,545	4,407	4,574	4,659	4,448
Pseudogenes	150	297	61	71	110	104	122	42
RNAs	100	99	112	162	99	23	77	82

*^*a*^Level indicates the completeness of the genome assembly*.

### Genome Diversity

To assess the genome diversity of the *V. diabolicus* subclade, the pangenome of the eight *V. diabolicus* subclade genomes (*V. diabolicus* Art-Gut C1 and CNCM I-1629, *V. antiquarius* 939 and EX25, and *V. alginolyticus* E0666, FF273, TS13, and V2) was determined by two methods: the clustering of homologous genes with *get_homologues* and the aligning of homologous genomic regions with Panseq. The *get_homologues* method reported 4,854 total and 3,422 core genes shared by all eight genomes. Thus, the pangenome consisted of 3,422 core genes (∼70.5% of the pangenome) and 1,422 accessory genes (∼29.5% of the pangenome). The Panseq method reported a 7,856,358 bp pangenome comprised of a 4,605,287 bp core genome (∼58.6% of the pangenome) and a 3,251,071 bp accessory genome (∼41.4% of the pangenome).

**FIGURE 4:**
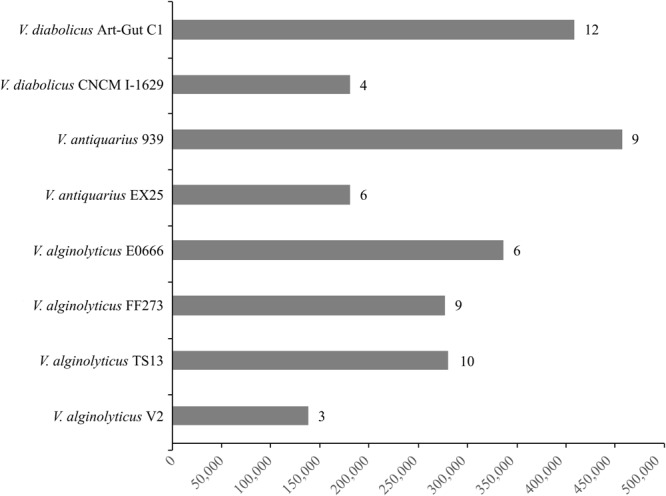
Novel regions and genomic islands (GIs). The cumulative length (bp) of novel genomic regions present in each *V. diabolicus* subclade genome. The bars are labeled with the number of probable GIs (i.e., novel genomic regions larger than 10 Kb) present in each genome.

The Panseq Novel Region Finder revealed that novel genomic regions totaling 2,295,535 bp accounted for the vast majority of the accessory genome: *V. diabolicus* Art-Gut C1 (415,117 bp), *V. diabolicus* CNCM I-1629 (183,383 bp), *V. antiquarius* 939 (464,398 bp), *V. antiquarius* EX25 (183,450 bp), *V. alginolyticus* E0666 (341,836 bp), *V. alginolyticus* FF273 (281,065 bp), *V. alginolyticus* TS13 (285,061 bp), and *V. alginolyticus* V2 (140,215 bp) (**Figure 4**). Further, among the novel genomic regions, probable genomic islands (GIs) greater than 10 Kb in length were prevalent: *V. diabolicus* Art-Gut C1 (12 GIs), *V. diabolicus* CNCM I-1629 (4 GIs), *V. antiquarius* 939 (9 GIs), *V. antiquarius* EX25 (6 GIs), *V. alginolyticus* E0666 (6 GIs), *V. alginolyticus* FF273 (9 GIs), *V. alginolyticus* TS13 (10 GIs), and *V. alginolyticus* V2 (3 GIs) (**Figure 4**). These GIs encoded a variety of proteins including hypothetical proteins, capsular polysaccharide biosynthesis proteins, lipopolysaccharide biosynthesis proteins, proteins related to carbohydrate and DNA metabolism, membrane transport proteins, phage-related proteins (phage baseplate proteins, tail proteins, tail fiber proteins, capsid proteins, endolysins, and integrases) and conjugative transfer proteins. However, these novel regions and GIs did not encode proteins with obvious habitat-specific functions. Rather, these novel regions encoded features thought to promote fitness in a range of habitats. In particular, a *V. alginolyticus* TS13 GI (26,181 bp) carried a widespread colonization island (WCI) with a tight-adherence (*tad*) locus containing 13 genes (*tadV-flp-rcpCAB-tadZABCDEFG*). Similarly, the alkyl hydroperoxide reductase proposed to scavenge endogenous hydrogen peroxide in the deep-sea (Hasan et al., 2015) was detected in all eight *V. diabolicus* strains.

### Subclade-Associated Genes

The search for genes ubiquitous in the *V. diabolicus* subclade (*N* = 8 strains) but absent from the other 41 strains, returned five genes (**Table 3**). All five were single-copy and highly conserved, producing gene and protein alignments with greater than 96% sequence identity. Two encoded hypothetical proteins (located on chromosome I) while the remaining three encoded a hydroxyectoine utilization dehydratase, an ornithine cyclodeaminase and a phosphoesterase (located on chromosome II).

**Table 3:** Description of open reading frames (ORFs) ubiquitous in *V. diabolicus* subclade strains (i.e., detected in *V. diabolicus* Art-Gut C1 and CNCM I-1629, *V. antiquarius* 939 and EX25, and *V. alginolyticus* E0666, FF273, TS13, and V2) but absent from the 41 other strains included in this study.

Accession^a^	Open reading frame^b^	Description	Length^c^	Sequence identity^d^
ACY49842.1	CP001805:76402-76626	Hypothetical protein	225 | 75	96.27 | 96.31
ACY50295.1	CP001805:552520-552717	Hypothetical protein	198 | 66	96.53 | 95.83
ACY53324.1	CP001806:741108-742082	Hydroxyectoine utilization dehydratase	975 | 325	99.14 | 99.14
ACY53325.1	CP001806:742097-743056	Ornithine cyclodeaminase	960 | 320	98.26 | 99.29
ACY53763.1	CP001806:1226999-1229245	Phosphoesterase	2,247 | 749	98.39 | 98.57

*^*a*^GenBank accession number in the type strain V. antiquarius EX25. ^*b*^Coordinates of the ORF in chromosome I (CP001805) or chromosome II (CP001806). ^*c*^Length of the gene (bp) and protein (aa). ^*d*^Average sequence identity of the gene and protein alignments between all V. diabolicus subclade genomes.*

### Metagenome Survey

Three subclade-associated coding sequences (hydroxyectoine utilization dehydratase, ornithine cyclodeaminase, and phosphoesterase) were selected as *V. diabolicus*-associated markers. When queried against the NCBI Metagenome Protein Database, proteins homologous to each were detected in more than 100 environmental metagenomes and the majority were categorized as marine metagenomes. For the hydroxyectoine utilization dehydratase query, the highest scoring hit showed 82% overage, 59% identity and a 2e-105 *E*-value. For the ornithine cyclodeaminase query, the highest scoring hit showed 96% overage, 49% identity and a 4e-96 *E*-value. For the phosphoesterase query, the highest scoring hit showed 46% overage, 23% identity and a 2e-06 *E*-value. Thus, all database hits were declared non-significant as the sequence identity was not comparable to the subclade protein alignments (i.e., greater than 95%).

### Potential Virulence

**FIGURE 5:**
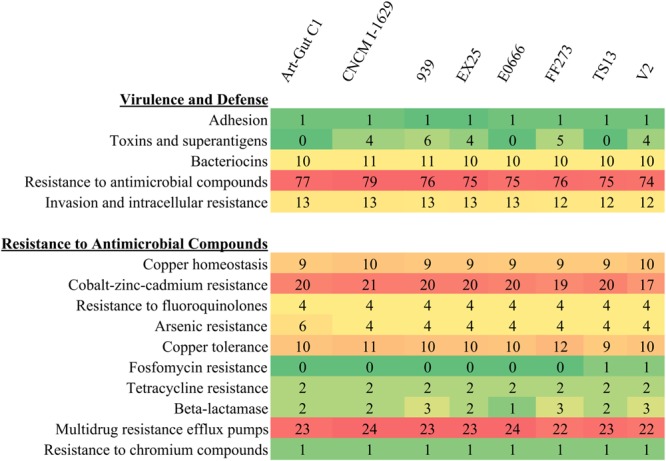
Heatmap of SEED subsystems. The number of SEED subsystem feature counts related to Virulence and Defense **(top)** with focus on subsystem feature counts related to Resistance to Antimicrobial Compounds **(bottom)**. Coloring correlates with the prevalence of specific features with rare features appearing green and abundant features appearing red.

A comparison of SEED subsystem features revealed a large repertoire of features associated with virulence and defense (**Figure 5**). Importantly, each of the eight *V. diabolicus* subclade genomes demonstrated a nearly identical subsystem profile. For example, the accessory colonization factor AcfA, assigned to the Adhesion subsystem, was present in all eight genomes. Similarly, 10–11 features assigned to the Bacteriocins and Ribosomally Synthesized Antibacterial Peptides subsystem were present in all genomes as part of a colicin V production cluster, and the 12–13 features assigned to the Invasion and Intracellular Resistance subsystem were present in all genomes as part of a suite of proteins with homology to a *Mycobacterium* virulence operon involved in protein synthesis. The exception was the Toxins and Superantigens subsystem where the number of features ranged from zero to six; however, when present, proteins assigned to this subsystem included the transcriptional activator ToxR, the transmembrane regulatory protein ToxS, one to two copies of the Zonula occludens toxin (Zot) and one to two copies of the accessory cholera enterotoxin (Ace).

Evaluation of subsystem features also revealed that 74–79 features were assigned to the Resistance to Antimicrobial Compounds subsystem. Further, when that subsystem was expanded into more specific subsystems (e.g., cobalt-zinc-cadmium resistance, tetracycline resistance and multidrug efflux pumps), all eight shared a nearly identical resistome (**Figure 5**). The resistome included features associated with resistance to toxic metals and metalloids (e.g., copper, cobalt, zinc, cadmium, chromium, and arsenic) and features associated with resistance to antibiotics (i.e., fluoroquinolones, fosfomycin, tetracycline, and penicillin). Additionally, the resistome included 22–24 efflux pumps per genome. These efflux pumps included pumps belonging to the multidrug and toxin extrusion (MATE) and resistance-nodulation-cell division (RND) families.

A phylogenetic analysis of proteins assigned to the β-lactamase subsystem (1–3 proteins per genome) revealed the presence of two distinct β-lactamases (**Supplementary Figure S2**). According to the comprehensive ARDB, these enzymes were identified as class A and class C β-lactamases associated with resistance to carbenicillin and cephalosporin, respectively. Similarly, the two FosA enzymes conferring resistance to fosfomycin, present as single copy genes in *V. alginolyticus* TS13 and V2, were identified as identical glutathione transferases, which confer resistance by catalyzing the addition of glutathione to fosfomycin.

### Antibiotic Susceptibility

A positive Cefinase test indicated that *V. antiquarius* 939 and *V. parahaemolyticus* RIMD2210633A produced β-lactamase. *V. antiquarius* 939 exhibited resistance to penicillin, ampicillin, and cephalothin, and susceptibility to carbenicillin. *V. parahaemolyticus* RIMD2210633 exhibited resistance to penicillin and cephalothin, intermediate susceptibility to ampicillin, and susceptibility to carbenicillin.

## Discussion

The taxonomy of the *Vibrio* genus is a story of rapid expansion. In the past four decades, the number of recognized species has grown from five (Bergey et al., 1974) to 139^4^. The taxonomy of the *Vibrio* genus is also a story of constant revision. In particular, species boundaries between members of the Harveyi clade (e.g., *V. campbellii, V. harveyi, V. rotiferianus, V. natriegens*, and *V. parahaemolyticus*) have proven especially difficult to delineate (Thompson et al., 2006). However, the advent of multilocus (Thompson et al., 2005, 2007; Sawabe et al., 2007, 2013; González-Escalona et al., 2008) and genome-scale phylogenetics (Thompson et al., 2009; Lin et al., 2010; Urbanczyk et al., 2013, 2014, 2016; Ke et al., 2017) has advanced our understanding of this once enigmatic clade. This study builds on previous work by clarifying the relatedness of two Harveyi clade species first isolated from the deep-sea: *V. diabolicus* and *V. antiquarius*.

Ultimately, questions surrounding the identity of *Vibrio* sp. 939 motivated genome sequencing and a subsequent genome-scale taxonomic analysis of 49 core and non-core members of the Harveyi clade. The initial examination of the draft genome in RAST indicated that the isolate was more closely related to *V. antiquarius*, motivating the *V. antiquarius* 939 assignment in GenBank. However, upon closer examination, a maximum-likelihood 49-genome phylogeny based on the concatenation of 1,109 homologous genes assigned the isolate to a monophyletic subclade comprised of two *V. diabolicus* strains (Art-Gut C1 and CNCM I-1629), two *V. antiquarius* strains (939 and EX25) and four *V. alginolyticus* strains (E0666, FF273, TS13, and V2).

Previous studies have proposed that soft species boundaries within the Harveyi clade can result from complex evolutionary scenarios, such as recombination, that obfuscate the phylogenetic signal (Fraser et al., 2007; Thompson et al., 2007). Seeing that complex evolutionary scenarios are difficult to represent in a phylogenetic tree (Huson and Bryant, 2006), a phylogenetic network was constructed to investigate the uncertainty of the species boundaries. The network revealed two groups of species that are separated by short branching: (1) *V. jasicida, V. harveyi, V. owensii*, and *V. campbellii*, and (2) *V. alginolyticus* and *V. diabolicus*. Additionally, a more pronounced pattern of reticulation between *V. alginolyticus* and *V. diabolicus* indicated that recombination obscured the phylogenetic signal between these two species. This short branching and pronounced reticulation could explain why the boundary between these two species was heretofore unresolved. Regardless, the subclades produced by the network analysis were well supported and mirrored the subclade assignments produced by the maximum-likelihood phylogeny.

The 95–96% ANI threshold is commonly applied to support species assignments (Richter and Rosselló-Móra, 2009; Varghese et al., 2015). This study showed that *V. diabolicus* (Art-Gut C1 and CNCM I-1629), *V. antiquarius* (939 and EX25), and *V. alginolyticus* (E0666, FF273, TS13, and V2) genomes shared greater than 97% ANI, which is a strong indication that they constitute the same species. In contrast, these genomes shared less than 92% ANI with representative *V. alginolyticus* genomes, confirming that they are distinct in comparison to their nearest phylogenetic neighbor. A similar study used the 97% ANI threshold to support the conclusion that *V. inhibens* is a heterotypic synonym of *V. jasicida* (Urbanczyk et al., 2016). In this study, the analysis of ANI values clearly showed that two *V. antiquarius* strains (939 and EX25) and four *V. alginolyticus* strains (E0666, FF273, TS13, and V2) are synonyms of *V. diabolicus*.

That the eight *V. diabolicus* strains described in this study were isolated from a range of sources (i.e., horse mackerel, seawater, sediment, dentex, oyster, artemia, and polychaete) collected from locations around the world (i.e., China, India, Greece, United States, East Pacific Rise, and Chile) suggests diverse physiological capabilities. Extensive genomic variability is thought to underlie a prokaryotic species’ ability to persist in a wide range of habitats (Cordero and Polz, 2014), and the genomic variability of *V. diabolicus*, evidenced in this study by a large accessory genome (relative to the number of genomes analyzed) and the presence of dozens of strain-specific GIs, may correlate with the species’ ability to persist in these varied habitats across the globe. For example, the ability to colonize substrates and form biofilms is critical to persistence, and the production of an adhesive Flp (fimbrial low-molecular-weight protein) pilus (encoded by a 13-gene *tad* locus found on a large GI in *V. alginolyticus* TS13) could facilitate the colonization of diverse habitats (Kachlany et al., 2001). However, the species appears to be conditionally rare as species-associated genetic markers, developed in this study, were not detected in the NCBI environmental metagenome database.

That the only known representatives of *V. antiquarius* (939 and EX25) are taxonomic synonyms of *V. diabolicus* represents a significant shift in the systematics of these bacteria as both *V. diabolicus* and *V. antiquarius* were long regarded as distinct species (Raguénès et al., 1997; Goudenège et al., 2014; Hasan et al., 2015). Yet the most striking synonym was *V. alginolyticus* E0666, which was isolated from spoiled horse mackerel associated with food poisoning (Cao et al., 2013) and later shown to be virulent in a murine model (Liu et al., 2013). The presence of virulence factors in *V. antiquarius* EX25, such as Ace and Zot, was described previously (Hasan et al., 2015). This study showed that the same virulence factors were present in other *V. diabolicus* subclade genomes, suggesting that virulence-associated genes could be ubiquitous across the species, which is not surprising given the prevalence of such features in closely related species (e.g., *V. alginolyticus* and *V. parahaemolyticus*). The species-wide conservation of these features supports the hypothesis that virulence-associated genes could play a fundamental role in the ecological setting (Hasan et al., 2015).

In the event that some *V. diabolicus* strains are virulent, intrinsic resistance to antibiotics could complicate treatment as the analysis of the species’ resistome revealed the presence of alleles and genes associated with resistance to fluoroquinolone, fosfomycin, tetracycline, and penicillin. The production of β-lactamase and resistance to penicillin, ampicillin, and cephalosporin was confirmed experimentally in *V. antiquarius* 939. The presence of numerous efflux pumps, including the MATE and RND families, could expand mechanisms of resistance. The ubiquity of these features is concerning given the species’ rarity and heretofore-perceived isolation, whereas widespread and progressive antibiotic resistance is a long-standing trend in *V. cholerae* (Garg et al., 2000; Uppal et al., 2017), *V. parahaemolyticus* (Baker-Austin et al., 2008; Devi et al., 2009), and *V. vulnificus* (Baker-Austin et al., 2009; Shaw et al., 2014).

## Conclusion

The results of this taxonomic and comparative genomic analysis revealed that *V. diabolicus* and *V. antiquarius* should be classified as the same species. As *V. diabolicus* was discovered and published (Raguénès et al., 1997; Goudenège et al., 2014) prior to *V. antiquarius* (Hasan et al., 2015), the latter is a taxonomic synonym. *V. alginolyticus* remains a distinct species, but four misidentified strains (E0666, FF273, TS13, and V2) are synonyms of *V. diabolicus*. The expansion of this species is ongoing as three new *V. diabolicus* genomes (FDAARGOS_96, LMG 3418, and FDAARGOS_105) were made publicly available during the review of this manuscript.

## Author Contributions

JWT, WN, RP, EA, and MS conceived and designed the research project. JWT, JJT, AM, LP, NE, and DNA conducted the genomic analyses and laboratory experiments. All authors contributed to data interpretation and the writing of the manuscript.

## Conflict of Interest Statement

The authors declare that the research was conducted in the absence of any commercial or financial relationships that could be construed as a potential conflict of interest.

## Footnotes

1 http://www.bacterio.net/vibrio.html

2 https://github.com/FelixKrueger/TrimGalore

3 http://tree.bio.ed.ac.uk/software/figtree/

4 http://www.bacterio.net/vibrio.html

## Supplementary Material

FIGURE S1 Phylogenetic tree of the *V. harveyi* clade. A maximum-likelihood tree representing 49 *V. harveyi* clade genomes was inferred from the concatenated alignment of 1,109 homologous genes. Node labels show the bootstrap support values. Nodes with strong support (>85) were highlighted in green while nodes with weak support (<85) were highlighted in red. Branch lengths represent the average number of substitutions per site. The tree was rooted to the outgroup comprised of *V. azureus* NBRC104587 and *V. sagamiensis* NBRC 104589.

FIGURE S2 Phylogenetic tree of β-lactamases. A maximum-likelihood tree showing the relatedness of 18 β-lactamases present in the eight *V. diabolicus* subclade genomes. Node labels show the bootstrap support values greater than 85. Branch lengths represent the average number of substitutions per site. The tree was rooted at the midpoint.

TABLE S1 List of the 49 Harveyi clade genomes included in this study. Genomes in bold font belong to the new *V. diabolicus* subclade described in this study.
