# Supplementary figures and images for: Comparative Genomic Analysis of Vibrio diabolicus and Six Taxonomic Synonyms: A First Look at the Distribution and Diversity of the Expanded Species
Source: Front Microbiol. 2018 Aug 15;9:1893. doi: 10.3389/fmicb.2018.01893 (PMC6104160; doi:10.3389/fmicb.2018.01893)

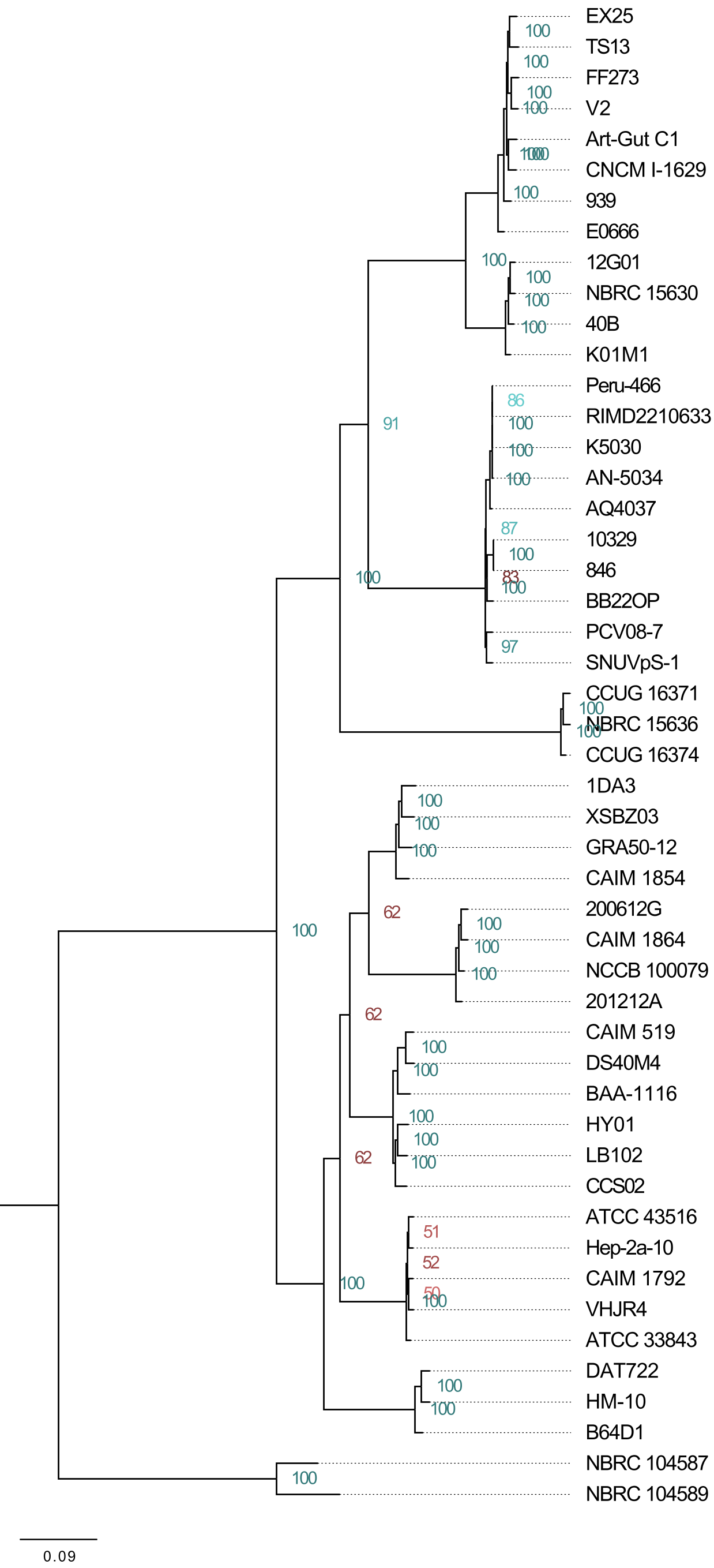

Supplement: FIGURE S1 — Phylogenetic tree of the V. harveyi clade. A maximum-likelihood tree representing 49 V. harveyi clade genomes was inferred from the concatenated alignment of 1,109 homologous genes. Node labels show the bootstrap support values. Nodes with strong support (>85) were highlighted in green while nodes with weak support (<85) were highlighted in red. Branch lengths represent the average number of substitutions per site. The tree was rooted to the outgroup comprised of V. azureus NBRC104587 and V. sagamiensis NBRC 104589. [file Image_1.TIF]

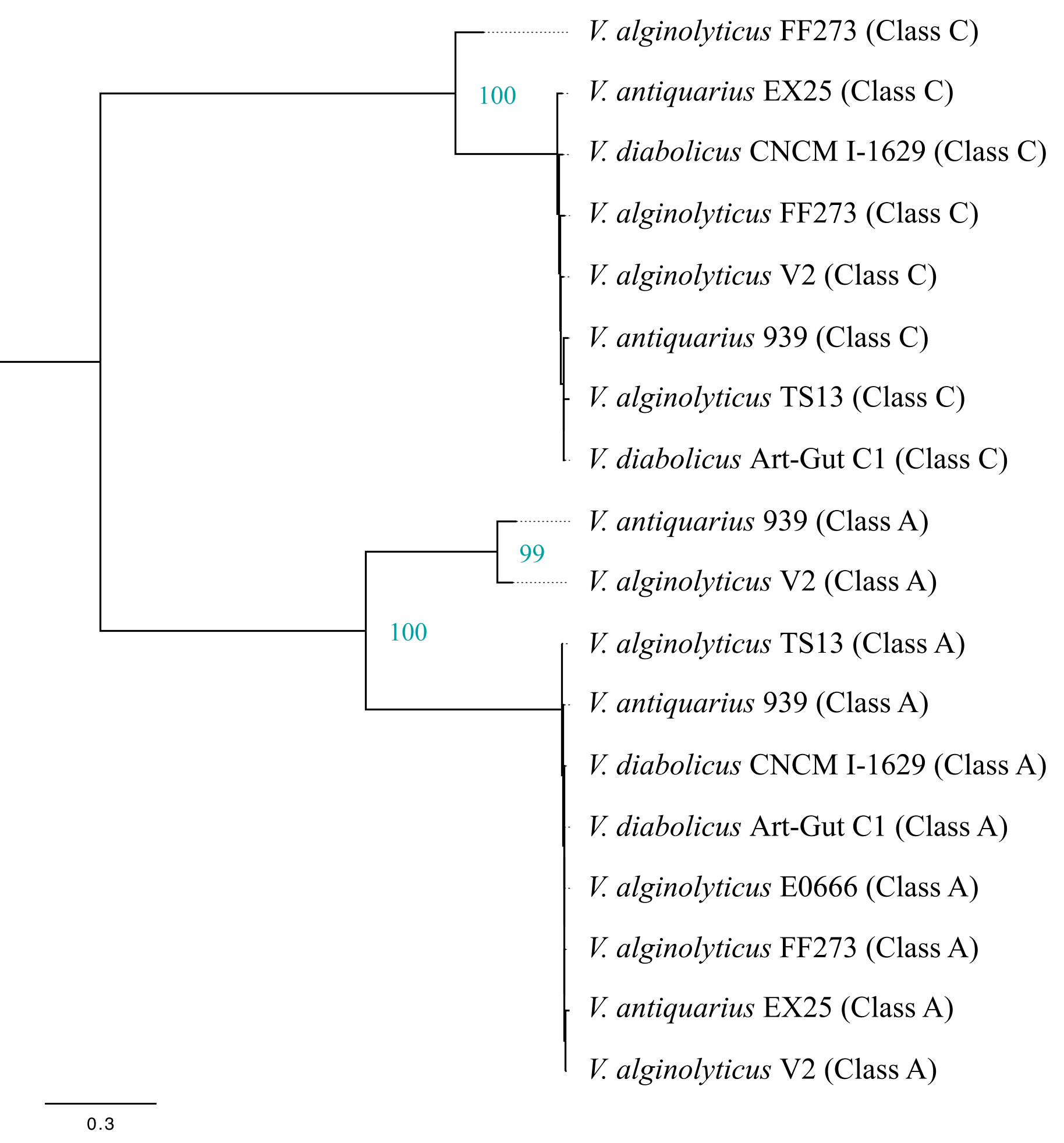

Supplement: FIGURE S2 — Phylogenetic tree of β-lactamases. A maximum-likelihood tree showing the relatedness of 18 β-lactamases present in the eight V. diabolicus subclade genomes. Node labels show the bootstrap support values greater than 85. Branch lengths represent the average number of substitutions per site. The tree was rooted at the midpoint. [file Image_2.TIF]
