# Supplementary material for: Comparative Genomic Analysis of Vibrio diabolicus and Six Taxonomic Synonyms: A First Look at the Distribution and Diversity of the Expanded Species
Source: Front Microbiol. 2018 Aug 15;9:1893. doi: 10.3389/fmicb.2018.01893 (PMC6104160; doi:10.3389/fmicb.2018.01893)
Supplement: TABLE S1 — List of the 49 Harveyi clade genomes included in this study. Genomes in bold font belong to the new V. diabolicus subclade described in this study. [file Table_1.docx]

**TABLE S1 |** List of the 49 Harveyi clade genomes included in this study. Genomes in bold font belong to the new *V. diabolicus* subclade described in this study.

| Isolate | Species | Source | Origin | Year | Accession | Reference |
| --- | --- | --- | --- | --- | --- | --- |
| 12G01 | *V. alginolyticus* | seawater | United States | 2006 | AAPS00000000 | Thompson et al., 2009 |
| 40B | *V. alginolyticus* | coral | Brazil | 2007 | ACZB00000000 | Thompson et al., 2009 |
| **E0666** | ***V. alginolyticus*** | **mackerel** | **China** | **2013** | **AMPD00000000** | **Cao et al., 2013** |
| **FF273** | ***V. alginolyticus*** | **seawater** | **United States** | **2006** | **MAKC00000000** | **Hehemann et al. 2016** |
| K01M1 | *V. alginolyticus* | pipefish | Germany | 2011 | CP017889, CP017890 | Wendling et al., 2017 |
| NBRC 15630 | *V. alginolyticus* | mackerel | China | 2013 | CP006718, CP006719 | Liu et al. 2015 |
| **TS13** | ***V. alginolyticus*** | **sediment** | **India** | **2012** | **JZWD00000000** | **unpublished** |
| **V2** | ***V. alginolyticus*** | **dentex** | **Greece** | **2007** | **LCSG00000000** | **Castillo et al., 2015** |
| **939** | ***V. antiquarius*** | **oyster** | **United States** | **2007** | **AOJB00000000** | **this study** |
| **EX25** | ***V. antiquarius*** | **seawater** | **East Pacific Rise** | **1999** | **CP001805, CP001806** | **Hasan et al., 2015** |
| NBRC 104587 | *V. azureus* | seawater | Japan | 2008 | BAOB00000000 | Yoshizawa et al., 2009 |
| BAA-1116 | *V. campbellii* | seawater | United States | 1993 | CP000789, CP000790, CP000791 | Lin et al., 2010 |
| CAIM 519 | *V. campbellii* | seawater | United States | 1971 | CP015863, CP015864, CP015865 | Thompson et al., 2007 |
| CCS02 | *V. campbellii* | barramundi | Australia | 2007 | BBKX00000000 | Cano-Gómez et al., 2011 |
| DS40M4 | *V. campbellii* | seawater | Africa | 2011 | AGIE00000000 | Dias et al., 2012 |
| HY01 | *V. campbellii* | shrimp | Thailand | 2004 | AAWP00000000 | Lin et al., 2010 |
| LB102 | *V. campbellii* | shrimp | India | 2006 | MWRX00000000 | Kumar et al., 2017 |
| **Art-Gut C1** | ***V. diabolicus*** | **artemia** | **Chile** | **2016** | **NCTH00000000** | **unpublished** |
| **CNCM I-1629** | ***V. diabolicus*** | **polychaete** | **East Pacific Rise** | **1991** | **CCKK00000000** | **Raguénès et al., 1997** |
| ATCC 33843 | *V. harveyi* | NA | United States | 1971 | CP009467, CP09468 | Wang et al., 2015 |
| ATCC 43516 | *V. harveyi* | shark | Bahamas | 1985 | CP014038, CP014039 | Pedersen et al., 1998 |
| CAIM 1792 | *V. harveyi* | shrimp | Mexico | 2005 | AHHQ00000000 | Espinoza-Valles et al., 2012 |
| Hep-2a-10 | *V. harveyi* | shrimp | United States | 2014 | MBTP01000000 | Moreno et al., 2017 |
| VHJR7 | *V. harveyi* | barramundi | Malaysia | 2009 | CAUO00000000 | Rasangan et al., 2012 |
| 200612G | *V. jasicida* | seawater | Japan | 2012 | BBKZ00000000 | Urbanczyk et al., 2014 |
| 201212A | *V. jasicida* | seawater | Japan | 2012 | BBLA00000000 | Urbanczyk et al., 2014 |
| CAIM 1864 | *V. jasicida* | lobster | New Zealand | 1999 | BAOG00000000 | Yoshizawa et a., 2012 |
| NCCB 100079 | *V. jasicida* | NA | Netherlands | 1924 | LNQW00000000 | Figge et al., 2011 |
| CCUG 16371 | *V. natriegens* | sediment | United States | NA | CP016347, CP016348 | unpublished |
| CCUG 16374 | *V. natriegens* | seawater | United States | NA | CP016351, CP016352 | unpublished |
| NBRC 15636 | *V. natriegens* | sediment | United States | 1961 | CP009977, CP009978 | Lee et al., 2016 |
| 1DA3 | *V. owensii* | coral | Brazil | 2007 | ACZC00000000 | Thompson et al., 2009 |
| CAIM 1854 | *V. owensii* | lobster | Australia | 2007 | JPRD00000000 | Urbanczyk et al., 2013 |
| GRA50-12 | *V. owensii* | algae | Taiwan | 2010 | BBPJ00000000 | Lin et al., 2015 |
| XSBZ03 | *V. owensii* | coral | China | 2013 | CP01959, CP019960 | unpublished |
| 10329 | *V. parahaemolyticus* | clinical | United States | 1998 | AFBW00000000 | González-Escalona et al., 2011 |
| 846 | *V. parahaemolyticus* | oyster | United States | 2015 | AOOX00000000 | Turner et al., 2016 |
| AN-5034 | *V. parahaemolyticus* | clinical | Bangladesh | 1998 | ACFO00000000 | Chen et al., 2011 |
| AQ4037 | *V. parahaemolyticus* | clinical | Maldives | 1985 | ACFN00000000 | Chen et al., 2011 |
| BB22OP | *V. parahaemolyticus* | clinical | Bangladesh | 1980 | CP003972, CP003973 | Jensen et al., 2013 |
| K5030 | *V. parahaemolyticus* | clinical | India | 2005 | ACKB00000000 | Chen et al., 2011 |
| PCV08-7 | *V. parahaemolyticus* | shrimp | Malaysia | 2008 | AOCL00000000 | Tiruvayipati et al., 2013 |
| Peru-466 | *V. parahaemolyticus* | clinical | Peru | 1996 | ACFM00000000 | Chen et al., 2011 |
| RIMD2210633 | *V. parahaemolyticus* | clinical | Thailand | 1996 | BA000031, BA000032 | Makino et al., 2003 |
| SNUVpS-1 | *V. parahaemolyticus* | clam | Korea | 2009 | AMRZ00000000 | Liu et al., 2013 |
| B64D1 | *V. rotiferianus* | seawater | China | 2015 | CP018311, CP018312 | unpublished |
| DAT722 | *V. rotiferianus* | mud crab | Australia | NA | AFAJ00000000 | Chowdhury et al., 2011 |
| HM-10 | *V. rotiferianus* | seahorse | China | 2015 | MKFT00000000 | unpublished |
| NBRC 104589 | *V. sagamiensis* | seawater | Japan | 2008 | BAOJ00000000 | Yoshizawa et al., 2010 |

**REFERENCES**

Cano-Gómez A., Høj L., Owens L., and Andreakis N. (2011). Multilocus sequence analysis provides basis for fast and reliable identification of *Vibrio harveyi*-related species and reveals previous misidentification of important marine pathogens. *Syst. Appl. Microbiol.* 34, 561–565. doi: 10.1016/j.syapm.2011.09.001

Cao Y., Liu X.F., Zhang H.L., Chen Y.J., and Hu C.J. (2013). Draft genome sequence of the human-pathogenic bacterium *Vibrio alginolyticus* E0666. *Genome Announc.* 1:e00686–13. doi:10.1128/genomeA.00686-13

Castillo D., D'Alvise P., Kalatzis P.G., Kokkari C., Middelboe M., Gram L., et al. (2015). Draft genome sequences of *Vibrio alginolyticus* strains V1 and V2, opportunistic marine pathogens. *Genome Announc.* 3:e00729–15–2. doi:10.1128/genomeA.00729-15

Chen Y., Stine O.C., Badger J.H., Gil A.I., Nair G.B., Nishibuchi M., et al. (2011). Comparative genomic analysis of *Vibrio parahaemolyticus*: serotype conversion and virulence. *BMC Genomics* 12:294. doi: 10.1186/1471-2164-12-294

Chowdhury R.P., Boucher Y., Hassan K.A., Paulsen I.T., Stokes H.W., and Labbate M. (2011). Genome sequence of *Vibrio rotiferiaus* strain DAT722. *J. Bacteriol.* 193, 3381–3382. doi: 10.1128/JB.05089-11

Dias G.M., Thompson C.C., Fishman B., Naka H., Haygood M.G., Crosa J.H., et al. (2012). Genome sequence of the marine bacterium *Vibrio campbellii* DS40M4, isolated from open ocean water. *J. Bacteriol.* 194, 904. doi: 10.1128/JB.06583-11

Espinoza-Valles I., Soto-Rodriguez S., Edwards R.A., Wang Z., Vora G.J., and Gomez-Gil B. (2012). Draft genome sequence of the shrimp pathogen *Vibrio harveyi* CAIM 1792. *J. Bacteriol*. 194, 2104. doi: 10.1128/JB.00079-12

Figge M.J., Robertson L.A., Ast J.C., and Dunlap P.V. (2011). Historical microbiology: revival and phylogenetic analysis of the luminous bacterial cultures of M. W. Beijerinck. *FEMS Microbiol. Ecol.* 78, 463–472. doi: 10.1111/j.1574-6941.2011.01177.x

González-Escalona N., Strain E.A., De Jesus A.J., Jones J.L., and DePaola A. (2011). Genome sequence of the clinical O4:K12 serotype *Vibrio parahaemolyticus* strain 10329. *J. Bacteriol*. 193, 3405–3406. doi: 10.1128/JB.05044-11

Hasan N.A., Grim C.J., Lipp E.K., Rivera I.N., Chun J., Haley B.J., et al. (2015). Deep-sea hydrothermal vent bacteria related to human pathogenic *Vibrio* species. *Proc. Natl. Acad. Sci. USA* 112, E2813–E2819. doi: 10.1073/pnas.1503928112

Hehemann J.H., Arevalo P., Datta M.S., Yu X., Corzett C.H., Henschel A., et al. (2016). Adaptive radiation by waves of gene transfer leads to fine-scale resource partitioning in marine microbes. *Nature Commun*. 7, 1286. doi: 10.1038/ncomms12860

Jensen R.V., DePasquale S.M., Harbolick E.A., Hong T., Kernell A.L., Kruchko D.H., et al. (2013). Complete genome sequence of prepandemic *Vibrio parahaemolyticus* BB22OP. *Genome Announc.* 1:e00002–12. doi:10.1128/genomeA.00002-12

Kumar S., Jangam A.K., Akhil V., Rajendran V., Katneni V.K., Sahaya Rajan J.J., Grover M., et al. (2017). Draft genome sequence of the luminescent strain *Vibrio campbellii* LB102, isolated from a black tiger shrimp (*Penaeus monodon*) broodstock rearing system. *Genome Announc.* 5: e00342–17–2. doi:[10.1128/genomeA.00342-17](https://dx.doi.org/10.1128%2FgenomeA.00342-17)

Lee H.H., Ostrov N., Wong B.G., Gold M.A., Khalil A., and Church G.M. (2016). *Vibrio natriegens*, a new genomic powerhouse. *bioRxiv*. doi:10.1101/058487

Lin B., Wang Z., Malanoski A.P., O'Grady E.A., Wimpee C.F., Vuddhakul V., et al. (2010). Comparative genomic analyses identify the *Vibrio harveyi* genome sequenced strains BAA-1116 and HY01 as *Vibrio campbellii*. *Environ. Microbiol. Rep.* 2, 81–89. doi: 10.1111/j.1758-2229.2009.00100.x

Lin L.C., Lin G.H., Tseng Y.H., and Yu M.S. (2015). Draft genome sequence of *Vibrio owensii* GRA50-12, isolated from green algae in the intertidal zone of eastern Taiwan. *Genome Announc.* 3:e01438–14–2. doi:10.1128/genomeA.01438-14

Liu M., and Chen S. (2013). Draft genome sequence of *Vibrio parahaemolyticus* V110, isolated from shrimp in Hong Kong. *Genome Announc.* 1:e00300-13. doi:10.1128/genomeA.00300-13.

Liu X.F., Cao Y., Zhang H.L., Chen Y.J., and Hu C.J. (2015). Complete genome sequence of *Vibrio alginolyticus* ATCC 17749. *Genome Announc.* 3: e01500–14–2. doi:[10.1128/genomeA.01500-14](https://dx.doi.org/10.1128%2FgenomeA.01500-14)

Makino K., Oshima K., Kurokawa K., Yokoyama K., Uda T., Tagomori K., et al. (2003). Genome sequence of *Vibrio parahaemolyticus*: a pathogenic mechanism distinct from that of *V. cholerae*. *Lancet* 361, 743–749. doi: 10.1016/S0140-6736(03)12659-1

Moreno E., Parks M., Pinnell L.J., Tallman J.J., and Turner J.W. (2017). Draft genome sequence of a *Vibrio harveyi* strain associated with Vibriosis in Pacific white shrimp (*Litopenaeus vannamei*). *Genome Announc.* 5:e01662–16–2. doi:10.1128/genomeA.01662-16

Pedersen K., Verdonck L., Austin B., Austin D.A., Blanch A.R., Grimont P.A.D., et al. (1998). Taxonomic evidence that *Vibrio carchariae* Grimes et al. 1985 is a junior synonym of *Vibrio harveyi* (Johnson and Shunk 1936) Baumann et al. 1981. *Int. J. Syst. Bacteriol.* 48, 749–758. doi: 10.1099/00207713-48-3-749

Raguénès G., Christen R., Guezennac J., Pignet P., and Barbier G. (1997). *Vibrio diabolicus* sp. nov., a new polysaccharide-secreting organism isolated from a deep-sea hydrothermal vent polychaete annelid, *Alvinella pompejana*. *Int. J. Syst. Bacteriol.* 47, 989–995. doi: 10.1099/00207713-47-4-989

Ransangan J., Lal T.M., and Al-Harbi A.H. (2012). Characterization and experimental infection of *Vibrio harveyi* isolated from diseased Asian seabass (*Lates calcarifer*). Malays. *J. Microbiol.* 8, 104–115. doi: 10.21161/mjm.03512

Tiruvayipati S., Bhassu S., Kumar N., Baddam R., Shaik S., Gurindapalli A.K., et al. (2013). Genome anatomy of the gastrointestinal pathogen, *Vibrio parahaemolyticus* of crustacean origin. *Gut Path*. 5, 37–45. doi: 10.1186/1757-4749-5-37.

Thompson F.L., Gomez-Gil B., Vasconcelos A.T., and Sawabe T. (2007). Multilocus sequence analysis reveals that *Vibrio harveyi* and *V. campbellii* are distinct species. *Appl. Environ. Microbiol.* 73, 4279–4285. doi: 10.1128/AEM.00020-07

Thompson C.C., Vicente A., Souza R.C., Vasconcelos A., Vesth T., Alves N., et al. 2009. Genomic taxonomy of Vibrios. *BMC Evol. Biol*. 9:258. doi: 10.1186/1471-2148-9-258

Turner J.W., Berthiaume C.T., Morales R., Armbrust E.V., and Strom M.S. (2016). Genomic evidence of adaptive evolution in emergent *Vibrio parahaemolyticus* ecotypes. *Elem. Sci. Anth.* 4, 117. [doi:10.12952/journal.elementa.000117](http://doi.org/10.12952/journal.elementa.000117)

Urbanczyk H., Ogura Y., and Hayashi T. (2013). Taxonomic revision of Harveyi clade bacteria (family *Vibrionaceae*) based on analysis of whole genome sequences. *Int. J. Syst. Evol. Microbiol.* 63, 2742–2751. doi: 10.1099/ijs.0.051110-0

Urbanczyk H., Ogura Y., and Hayashi T. (2014). Contrasting inter- and intraspecies recombination patterns in the “Harveyi Clade” *Vibrio* collected over large spatial and temporal scales. *Genome Biol. Evol*. 7, 71–80. doi: 10.1093/gbe/evu269

Wang Z., Hervey W.J., Kim S., Lin B., and Vora G.J. (2015). Complete Genome Sequence of the Bioluminescent Marine Bacterium *Vibrio harveyi* ATCC 33843 (392 [MAV]). *Genome Announc.* 3:e01493–14–2. doi:10.1128/genomeA.01493-14

Wendling C.C., Piecyk A., Refardt D., Chibani C., Hertel R., Liesegang H., et al. 2017. Tripartite species interaction: eukaryotic hosts suffer more from phage susceptible than from phage resistant bacteria. *BMC Evol. Biol*. 17:98. doi: 10.1186/s12862-017-0930-2

Yoshizawa S., Wada M., Kita-Tsukamoto K., Ikemoto E., Yokota A., and Kogure K. (2009). *Vibrio azureus* sp. nov., a luminous marine bacterium isolated from seawater. *Int. J. Syst. Evol. Microbiol.* 59, 1645–1649. doi: 10.1099/ijs.0.004283-0

Yoshizawa S., Wada M., Yokota A., and Kogure K. (2010). *Vibrio sagamiensis* sp. nov., luminous marine bacteria isolated from sea water. *J. Gen. Appl. Microbiol*. 56, 499–507. doi: 10.2323/jgam.56.499

Yoshizawa S., Tsuruya Y., Fukui Y., Sawabe T., Yokota A., Kogure K., et al. (2012). *Vibrio jasicida* sp. nov., a member of the Harveyi clade, isolated from marine animals (packhorse lobster, abalone and Atlantic salmon). *Int. J. Syst. Evol. Microbiol*. 62, 1864–1870. doi: 10.1099/ijs.0.025916-0
